# Paeonol Ameliorates Benign Prostatic Hyperplasia via Suppressing Proliferation and NF-κB—In Silico and Experimental Studies

**DOI:** 10.3390/ph18091322

**Published:** 2025-09-03

**Authors:** Han-Young Lee, Min-Seong Lee, Byung-Cheol Lee

**Affiliations:** Department of Clinical Korean Medicine, Graduate School, Kyung Hee University, 26 Kyungheedae-ro, Dongdaemun-gu, Seoul 02447, Republic of Korea; hanyoung2357@gmail.com

**Keywords:** paeonol, benign prostatic hyperplasia, dihydrotestosterone, proliferation, NF-κB, Akt1

## Abstract

**Background/Objectives:** Benign prostatic hyperplasia (BPH) is a prevalent urological disorder in aging men, characterized by the enlargement of prostate epithelial and stromal cells, which leads to lower urinary tract symptoms. Paeonol, a bioactive compound derived from Moutan Cortex (*Paeonia suffruticosa*), exhibits multiple pharmacological properties; however, its therapeutic potential in BPH remains unclear. This study aimed to elucidate the mechanisms of paeonol in BPH treatment using network pharmacology and in vivo experiments. **Methods:** Network pharmacology and molecular docking were conducted to identify potential targets of paeonol against BPH. For the in vivo study, testosterone-induced BPH rat models were employed, and efficacy was evaluated through prostate weight assessment, histological examination, and the quantitative real-time polymerase chain reaction (qRT-PCR) analysis of prostate tissues. **Results:** In silico analysis revealed key signaling pathways involved in apoptosis, proliferation, phosphatidylinositol 3-kinase (PI3K)–protein kinase B (Akt), and inflammation. Paeonol administration significantly reduced prostate weight, volume, and histological hyperplasia in BPH rats. qRT-PCR analysis demonstrated that paeonol may suppress dihydrotestosterone production by inhibiting 5α-reductase 2 (5AR2) and the androgen receptor (AR), while also downregulating local growth factors, alpha serine/threonine-protein kinase (Akt1), nuclear factor-κB (NF-κB), and glutathione reductase (GR) expression. **Conclusions:** These findings provide novel insights into the multitargeted therapeutic potential of paeonol in BPH by inhibiting 5AR and AR and suppressing proliferation via NF-κB and Akt pathway modulation.

## 1. Introduction

Benign prostatic hyperplasia (BPH) is a common condition in older men, characterized by prostate enlargement and urinary dysfunction. BPH adversely affects quality of life, and its prevalence increases with age, affecting 40–50% of men aged 50–60 years and up to 90% of those aged over 80 years [1]. Histologically, BPH involves abnormal growth of glandular epithelium and stromal cells, driven by hormonal imbalances between androgens and estrogens and elevated growth factor levels [2]. As the prostate enlarges, it compresses the urethra, causing lower urinary tract symptoms (LUTS), such as reduced urinary flow, bladder outlet obstruction, incomplete emptying, nocturia, and frequent urination [3,4]. Despite the high prevalence and significant impact on quality of life, the underlying mechanisms of BPH remain incompletely understood. Multiple factors, including an imbalance between cell proliferation and apoptosis, hormonal dysregulation, oxidative stress, and inflammation, are implicated in BPH progression, presenting several therapeutic targets [4,5]. Current pharmacological treatments for BPH include α-adrenoceptor blockers, which alleviate LUTS by relaxing smooth muscle, and 5α-reductase (5AR) inhibitors, which reduce prostate size by suppressing DHT production and subsequent cell proliferation [6,7]. However, long-term use of these medications may lead to side effects, including insomnia, erectile dysfunction, and increased risk of prostate cancer [8].

**Figure 1:**
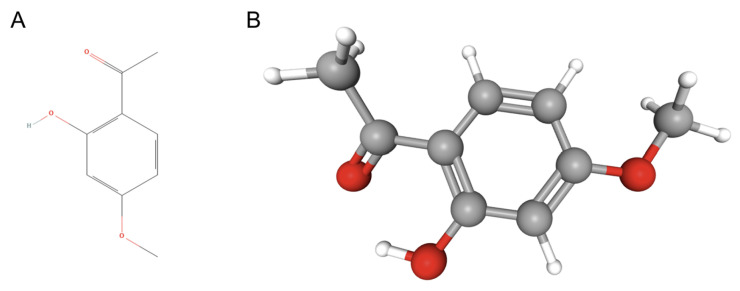
Molecular configuration of paeonol. (**A**) Two-dimensional representation from PubChem. (**B**) Three-dimensional representation from PubChem (accessed on 24 August 2025). The red color represents oxygen atoms.

Given the limitations of conventional therapies, the development of new therapeutic agents for BPH has gained growing attention [9,10,11]. Moutan Cortex, derived from the root bark of *Paeonia suffruticosa*, has been studied for its antioxidative, anti-inflammatory, antidiabetic, and antiproliferative effects [12]. Paeonol, its primary active compound, has been widely investigated for its anti-inflammatory properties such as inhibition of interleukin (IL)-1, IL-6, cyclooxygenase-2 (COX-2), TNF-α, and NF-κB activation [13] (Figure 1). Its anti-cardiovascular effects include suppression of endothelial cell apoptosis and vascular smooth muscle cell proliferation by regulating intercellular adhesion molecules and vascular cell adhesion molecules and inhibiting NF-κB signaling, endoplasmic reticulum stress, and reactive oxygen species (ROS), and its anti-tumor effects are elaborated through modulation of apoptosis and cell proliferation [13,14]. However, the therapeutic effects of paeonol in the prostate remain unexplored. Although Xu et al. [15] reported anti-prostate cancer effects of paeonol through modulating apoptosis via capspase-dependent and PI3K/Akt pathways in DU145 cell lines, its efficacy in BPH remains unexplored to the best of our knowledge. This study aimed to evaluate the therapeutic efficacy and elucidate the underlying molecular mechanisms of paeonol against BPH through in silico and in vivo methods. Prior to animal experiment, we analyzed protein–protein interaction (PPI) networks and identified hub genes and pathways using network pharmacology. Potential therapeutic targets of paeonol in BPH were further evaluated through molecular docking. Therapeutic effects of paeonol were subsequently validated in testosterone propionate (TP)-induced BPH rat models, and the underlying molecular mechanisms involving various factors contributing to BPH progression were comprehensively investigated through prostate organ weight evaluation, histological examination, and quantitative real-time polymerase chain reaction (qRT-PCR) analysis. To the best of our knowledge, this study presents the first evidence of the multifaceted therapeutic effects of paeonol in BPH development.

## 2. Results

### 2.1. Network Pharmacology Analysis Results

#### 2.1.1. Identification and Network Analysis of Molecular Targets

**Figure 2:**
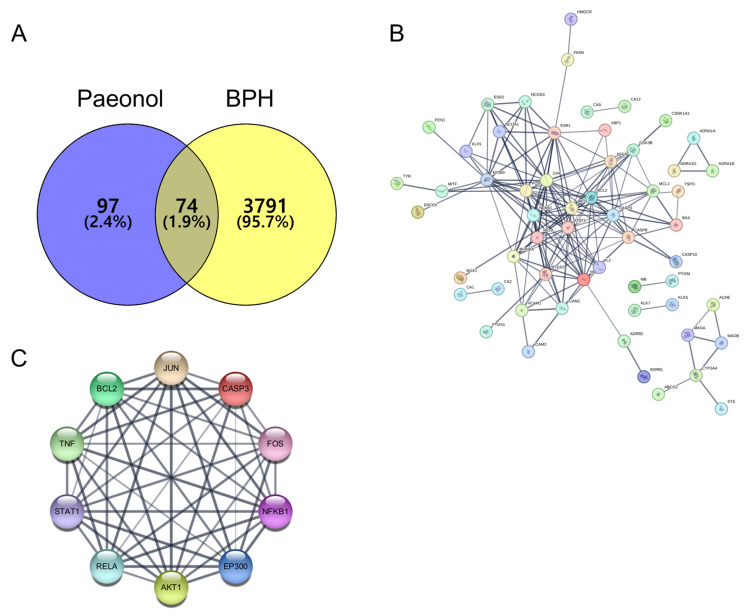
In silico PPI analysis of paeonol against BPH. (**A**) Intersections between paeonol and the BPH targets are depicted in a Venn diagram; (**B**) mapping of paeonol-BPH target interactions using the STRING database; (**C**) identification of the top 10 central hub genes in the PPI network. PPI, protein–protein interaction. In the Venn diagram (**A**), each color represents a different set of genes. In the STRING network map (**B**), the color of each node indicates the functional group or association of the protein, and the color of the edges indicates the type of evidence for the interaction. In the PPI network (**C**), each node represents a protein, and the color of the nodes indicates the functional module or cluster to which the protein belongs.

To assess the therapeutic potential of paeonol in the treatment of BPH, we predicted its molecular targets using various online databases. The drug-like properties of paeonol were evaluated using SwissADME (http://www.swissadme.ch/, accessed on 13 July 2023), which confirmed its compliance with Lipinski’s rule of five. Among 171 target proteins of paeonol and 3865 BPH-related proteins, 74 overlapping targets were identified, indicating potential molecular targets of paeonol in the treatment of BPH (Figure 2A). To gain deeper insights into the functional interactions among the 74 shared targets and to elucidate the mechanisms underlying paeonol’s action in BPH, a PPI network was constructed (Figure 2B). Further analysis identified the top 10 hub genes based on their degree of connectivity within the network, including alpha serine/threonine-protein kinase (Akt1), B-cell lymphoma 2 (BCL-2), caspase 3 (CASP3), tumor necrosis factor (TNF), and nuclear factor-κB (NF-κB), providing insight into the key proteins that may mediate the therapeutic effects of paeonol in BPH (Figure 2C).

#### 2.1.2. Enrichment Analysis of the Gene Ontology (GO) and Kyoto Encyclopedia of Genes and Genomes (KEGG) Pathways

**Figure 3:**
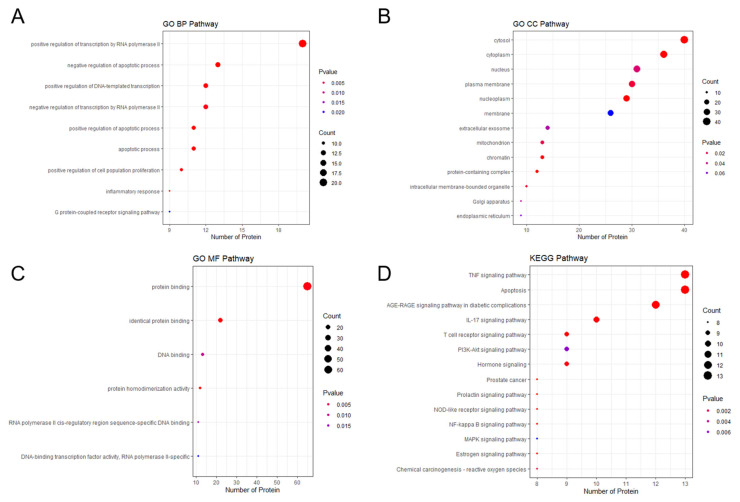
Enrichment analysis of pathways related to BPH (**A**) GO terms for biological processes; (**B**) GO terms for cellular components; (**C**) GO terms for molecular functions; (**D**) KEGG pathway analysis. GO, Gene Ontology. KEGG, Kyoto Encyclopedia of Genes and Genomes.

GO analysis showed relevant pathways such as apoptotic process and inflammatory response, cytosol and cytoplasm, and protein binding (Figure 3A–C). KEGG enrichment analysis revealed TNF signaling, apoptosis, advanced glycation end-product receptor for advanced glycation end-product (AGE–RAGE) signaling, IL-17 signaling, phosphoinositide 3-kinase (PI3K)–Akt signaling, and NF-kB signaling pathways (Figure 3D).

#### 2.1.3. Analyzing Paeonol’s Interaction with BPH-Related Proteins via Molecular Docking

**Figure 4:**
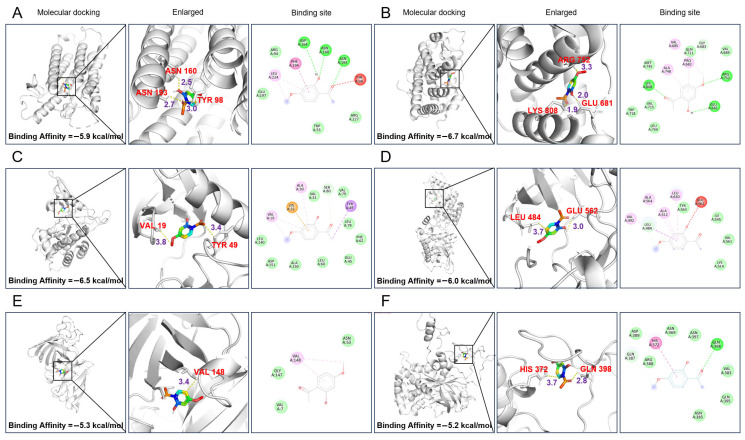

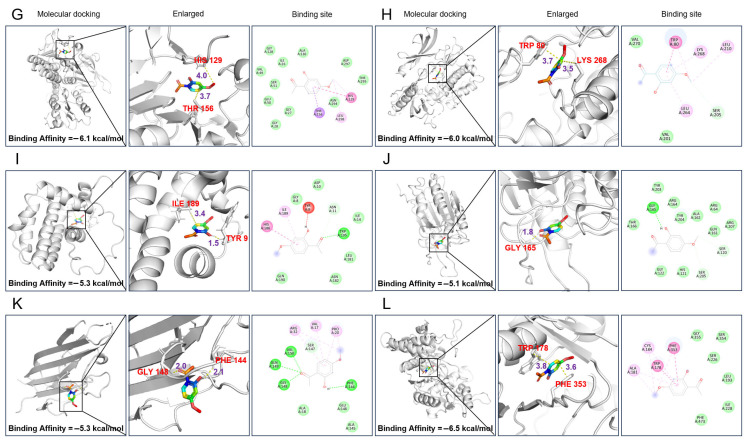
Docking of paeonol with several target proteins associated with BPH progression. (**A**) 5AR2; (**B**) AR; (**C**) TGF-β1; (**D**) FGF-1; (**E**) SOD1; (**F**) CAT; (**G**) GR; (**H**) Akt1; (**I**) BCL-2; (**J**) CASP3; (**K**) TNF; (**L**) NF-κB. The molecular docking images show the interactions between Paeonol and each protein. The green dotted lines represent hydrogen bonds, and the purple dotted lines represent hydrophobic interactions. Binding Affinity is a score (in kcal/mol) that predicts the strength with which Paeonol binds to each protein, where a lower value indicates a stronger bond.

We conducted molecular docking studies using the PyRx software (ver 0.8) to predict the binding affinity of paeonol to several key proteins associated with BPH progression. The docking targets included 5AR2 (−5.9 kcal/mol, PDB ID: 7BW1) and androgen receptor (AR) (−6.7 kcal/mol, PDB ID: 5JJM) regarding dihydrotestosterone (DHT) production; transforming growth factor beta-1 (TGF-β1) (−6.5 kcal/mol, PDB ID: 3GXL) and fibroblast growth factor 1 (FGF-1) (−6.0 kcal/mol, PDB ID: 1FGK), which are related to proliferation; superoxide dismutase 1 (SOD1) (−5.3 kcal/mol, PDB ID: 1OZU), catalase (CAT) (−5.2 kcal/mol, PDB ID: 1F4J), and glutathione reductase (GR) (−6.1 kcal/mol, PDB ID: 1GSN) regarding oxidative stress; Akt1 (−6.0 kcal/mol, PDB ID: 3O96), BCL-2 (−5.3 kcal/mol, PDB ID: 1G5M) and CASP3 (−5.1 kcal/mol, PDB ID: 3DEI) regarding apoptosis; and TNF (−5.3 kcal/mol, PDB ID: 5UUI) and NF-κB (−6.5 kcal/mol, PDB ID: 6POZ), regarding inflammation. The binding energies for all proteins were below −5 kcal/mol, indicating a strong binding affinity between paeonol and these targets. In addition, paeonol established hydrogen bonds with key residues within the binding pockets, such as ASP164/ASN160/ASN193 in 5AR2 and GLU681/ARG752/LYS808 in AR, as well as GLN398/ASN385 in CAT, GLU562/TYR563 in FGF-1, and PHE353/TRP178/CYS184 in NF-κB. These interactions suggest that paeonol may exert stabilizing effects through both hydrogen bonding and hydrophobic contacts with critical residues of BPH-related targets. The detailed docking results are shown in Table 1 and Figure 4.

### 2.2. In Vivo Experiment

#### 2.2.1. Effects of Paeonol on Body Weight and Prostate in BPH

**Figure 5:**
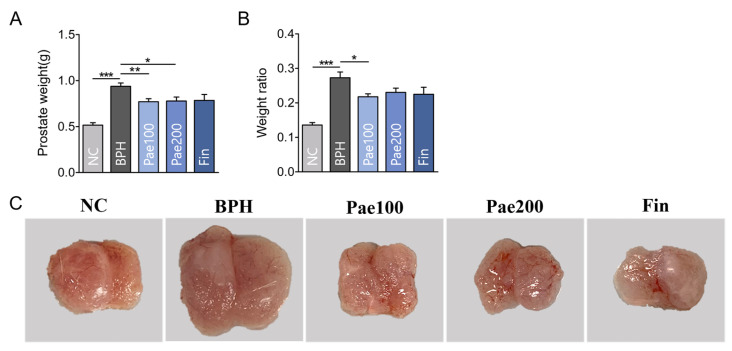
Effects of paeonol on prostate: (**A**) Prostate weight; (**B**) ratio of prostate weight to body weight ×100; (**C**) representative image of collected prostate. Data are represented as mean ± SEM (n = 5). * *p* < 0.05, ** *p* < 0.01, *** *p* < 0.001. NC, normal control group; BPH, TP-induced BPH group; Pae100, Pae-treated BPH group (100 mg/kg, p.o.); Pae200, Pae-treated BPH group (200 mg/kg, p.o.); Fin, Finasteride-treated BPH group.

This study aimed to evaluate the effects of a 30-day treatment regimen in testosterone-induced BPH rat model with oral administration of paeonol. At the end of the study, prostate weight, and relative prostate weight were significantly higher in the BPH group than in the normal control (NC) group. Treatment with paeonol significantly reduced both prostate weight and its relative weight ratio (Figure 5A,B). Representative images of prostate samples from each group showed noticeably reduced prostate size in the paeonol-treated groups (Figure 5C).

#### 2.2.2. Effects of Paeonol on Prostate Tissue Morphology

**Figure 6:**
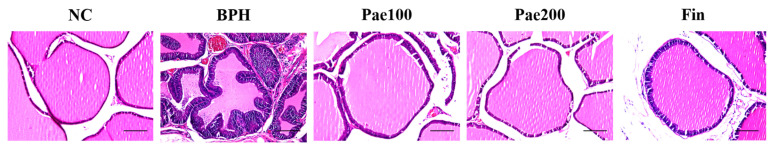
Histological changes in prostate tissues (hematoxylin and eosin (H&E) staining, ×200). Representative images showing morphological alterations across groups. Bar indicates 100 µm. NC (normal control): Round acinar glands with a single layer of cylindrical epithelial cells and no stromal proliferation or fibrosis. BPH (testosterone-induced): Pronounced glandular hyperplasia with elongated and irregular acini, multilayered epithelial cells, and severe stromal proliferation with fibrosis. Pae100 (Paeonol 100 mg/kg): Partial restoration of normal morphology with reduced epithelial proliferation and stromal thickening. Pae200 (Paeonol 200 mg/kg): Dose-dependent improvement with rounded acini, cuboidal epithelial cells, and markedly decreased stromal proliferation and fibrosis. Fin (Finasteride 1 mg/kg): Comparable reduction in hyperplasia, showing improvement similar to paeonol treatment.

Prostate tissue histomorphology was evaluated using hematoxylin and eosin (H&E) staining. In the NC group, the acinar glands retained a round shape with normal cylindrical epithelial cells, and no proliferation or fibrosis of the connective tissue was observed. By contrast, the BPH group exhibited significant glandular proliferation, with acinar epithelial cells assuming oval or linear shapes and irregular distribution with several folds. The connective tissues exhibited severe cellular proliferation and fibrosis. In paeonol-treated groups, the acinar glands appeared more rounded with less atrophy than in the BPH group. The acinar epithelial cells maintained a cuboidal shape with more regular distribution, and the connective tissue showed milder proliferation and reduced fibrosis (Figure 6).

#### 2.2.3. Effects of Paeonol on Serum Testosterone Levels

**Figure 7:**
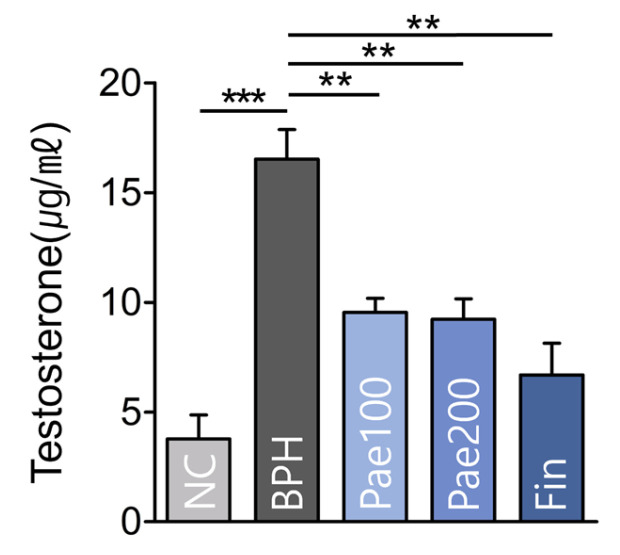
Serum testosterone levels of each experimental group. Data are represented as mean ± SEM (n = 5). ** *p* < 0.01, *** *p* < 0.001. NC, normal control group; BPH, TP-induced BPH group; Pae100, Pae-treated BPH group (100 mg/kg, p.o.); Pae200, Pae-treated BPH group (200 mg/kg, p.o.); Fin, Finasteride-treated BPH group.

To investigate the hormonal effects of paeonol on BPH treatment, serum testosterone levels were analyzed. The BPH group showed a significant increase compared to the NC group, whereas paeonol treatment significantly reduced serum testosterone levels (Figure 7).

#### 2.2.4. Effects of Paeonol on Gene Expression in the Prostate Tissue

**Figure 8:**
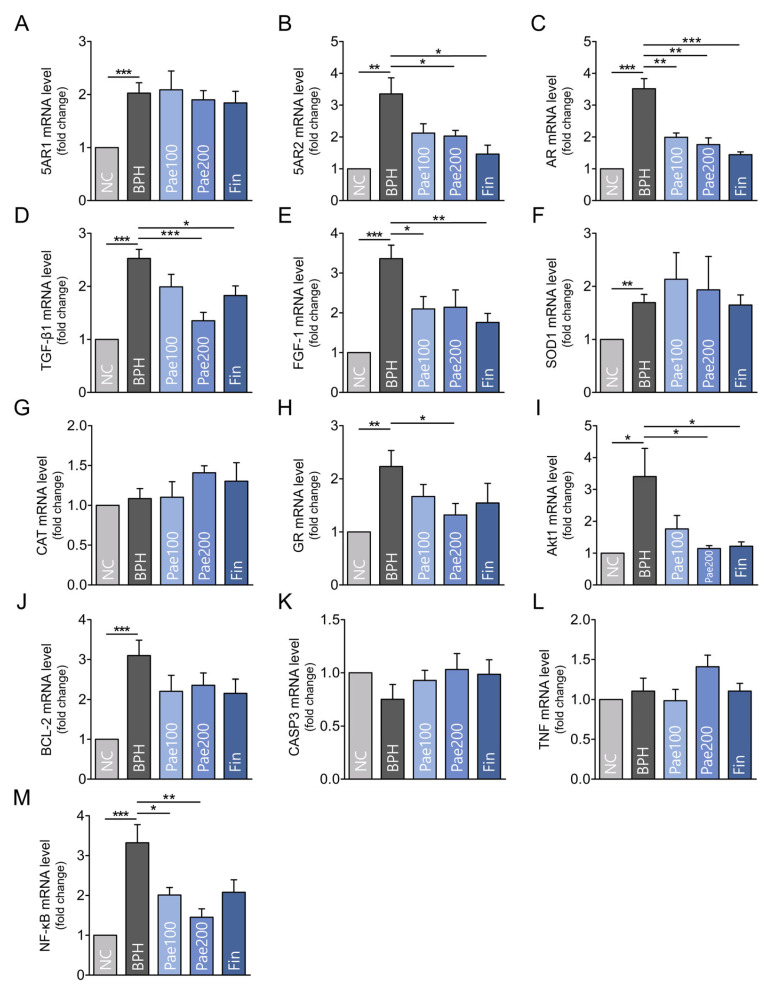
Effects of paeonol on gene expression in prostate tissues. Relative mRNA expression levels of BPH-related genes determined by qRT-PCR. (**A**) 5AR1; (**B**) 5AR2; (**C**) AR; (**D**) TGF-β1; (**E**) FGF-1; (**F**) SOD1; (**G**) CAT; (**H**) GR; (**I**) Akt1; (**J**) BCL-2; (**K**) CASP3; (**L**) TNF; (**M**) NF-κB. Androgen signaling: 5AR2 and AR were significantly upregulated in the BPH group compared with NC, and markedly downregulated by paeonol and finasteride. Growth factors: TGF-β1 and FGF-1 were elevated in BPH and significantly suppressed by paeonol. Oxidative stress regulator: GR expression was increased in BPH but reduced in the Pae200 group. Inflammatory pathway: NF-κB was significantly suppressed by paeonol in a dose-dependent manner, while TNF showed no significant changes. Other targets: Expression of 5AR1, SOD1, CAT, BCL-2, and CASP3 showed minimal or insignificant changes. Data are represented as mean ± SEM (n = 5). * *p* < 0.05, ** *p* < 0.01, *** *p* < 0.001. NC, normal control group; BPH, TP-induced BPH group; Pae100, Pae-treated BPH group (100 mg/kg, p.o.); Pae200, Pae-treated BPH group (200 mg/kg, p.o.); Fin, Finasteride-treated BPH group.

Gene expression levels of 5AR1, 5AR2, AR, TGF-β1, FGF-1, SOD1, GR, Akt1, BCL-2, CASP3, TNF, and NF-κB in the BPH group were significantly upregulated compared to the NC group, while CAT, CASP3, and TNF levels remained insignificant between groups (Figure 8). 5AR2 and AR expression levels were significantly inhibited in the paeonol group compared to the BPH group (Figure 8B,C). Regarding growth factors, paeonol significantly suppressed TGF-β1 and FGF-1 expression (Figure 8D,E). Pae200 group showed significantly downregulated the expression of GR and Akt1 (Figure 8H,I). Paeonol groups showed suppressed NF-κB levels in a dose-dependent manner (Figure 8M).

#### 2.2.5. Effects of Paeonol on Kidney and Liver Functions

**Figure 9:**
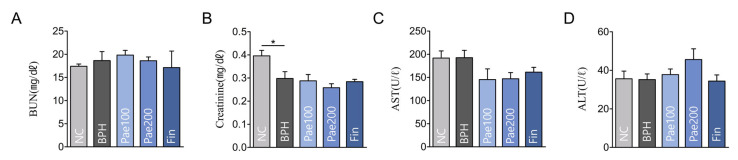
BUN, Creatinine, AST, and ALT levels in each experimental group (**A**) BUN levels; (**B**) Creatinine levels; (**C**) AST levels; (**D**) ALT levels. Data are represented as mean ± SEM (n = 5). * *p* < 0.05. NC, normal control group; BPH, TP-induced BPH group; Pae100, Pae-treated BPH group (100 mg/kg, p.o.); Pae200, Pae-treated BPH group (200 mg/kg, p.o.); Fin, Finasteride-treated BPH group. BUN, Blood urea nitrogen; AST, Aspartate transaminase; ALT, Alanine transaminase.

Renal and hepatic functions were assessed by measuring the serum blood urea nitrogen (BUN), creatinine, aspartate aminotransferase (AST), and alanine aminotransferase (ALT) levels. Creatinine levels significantly decreased in the BPH group compared to those in the NC group, although all values remained within the normal range. All serum markers showed no significant difference in the experimental groups (Figure 9).

## 3. Discussion

BPH, which is characterized by an overgrowth of prostatic stromal and epithelial cells, is a nonmalignant condition caused by an imbalance between cell apoptosis and proliferation [16]. This enlargement affects quality of life by causing LUTS, and in severe cases, BPH leads to serious complications such as urinary retention, bladder decompensation, and upper urinary tract infections [4,7]. Although BPH is common in aging men, its pathogenesis remains unclear, hindering the development of effective treatments [16]. Current theories involve epithelial–mesenchymal interactions, inflammation, hormonal imbalances, apoptosis, growth factors, and oxidative stress [5,17,18]. 5AR inhibitors, which is the only pharmacological option to prevent BPH progression, are associated with adverse effects such as erectile dysfunction and diminished libido, thus necessitating development of other therapeutic agents [8]. Paeonol, an active compound of Moutan Cortex, has elaborated anti-inflammatory, anti-proliferative, and anti-oxidative aspects [13,14]. Nonetheless, previous studies on its therapeutic effects in the prostate are limited to DU145 cell lines [15]. This study presents the first evidence of paeonol as a potential therapeutic agent against BPH.

While multiple factors are involved in BPH progression, controlling DHT production is considered the most crucial factor [7]. As binding of DHT with androgen receptor induces increased synthesis of androgen-related proteins and growth factors and inhibits apoptosis, inhibition of DHT and its binding to the androgen receptor is considered a piotal therapeutic target in BPH treatment [16]. Of the 5AR isoenzymes, types 1 and 2 are most relevant in BPH, with type 2, the predominant prostatic 5AR located on the nuclear membrane of stromal and epithelial cells in prostate, playing a major role in DHT production [19]. Paeonol significantly inhibited 5AR2 and AR expressions in the prostate tissue of the TP-induced BPH rat model. Furthermore, paeonol significantly reduced enlarged prostate size, followed by inhibition of prostatic glandular proliferation in histological images. Collectively, these results suggest that paeonol may prevent DHT production and subsequent enlargement of the prostate tissue Via inhibition of 5AR2 and AR. Further direct evaluation of DHT level is warranted in future studies.

BPH is described as a disruption of prostatic homeostasis between cell proliferation and apoptosis [16]. Growth factors, such as the FGFs, TGF-β family, and epidermal growth factors, are involved in prostate growth and the regulation of proliferation [20]. While previous studies suggested anti-proliferative effects of paeonol in cardiovascular diseases [21] and in silico results identified AGE-RAGE and PI3K-Akt pathways as relevant and showed high binding affinity with FGF-1 and TGF-β1 with values over −6 kcal/mol, no previous studies have investigated inhibitory effects of paeonol on local growth factors in BPH. Among many growth factors, FGFs play dominant role as they regulate epithelial proliferation, differentiation, angiogenesis, and apoptosis [22]. While FGFs are upregulated in BPH prostate tissue, FGF activation is associated with multiple factors, such as androgens, TGF-β, hypoxic damage, and inflammatory cytokines. Paeonol significantly inhibited FGF-1 expression in the prostate tissue, suggesting ameliorative effects of paeonol on epithelial cell proliferation. Suppressive effects of paeonol on FGF-1 expression warrants further study on whether paeonol acts as a direct inhibitor of FGF/FGFR or if it is an indirect consequence of suppression of androgens or inflammation. Interestingly, the effects of TGF-β1 in BPH are related to dysfunction of luminal epithelial barrier of the prostate, leading to fibrosis and inflammation, stromal cell differentiation and stimulation of extracelluar matrix (ECM) production rather than cell proliferation itself, and it is considered a promising therapeutic target in BPH [18,23]. Paeonol markedly inhibited TGF-β1 levels in TP-induced BPH prostate tissue, allowing reduced ECM in BPH. Taken together, our study provides the first evidence that paeonol attenuates prostate cell proliferation in a BPH rat model by downregulating FGF-1 and TGF-β1 expression, and this is supported by histological images showing mitigation in proliferated glands and fibromuscular stroma.

Reduced apoptosis is another crucial element in BPH progression, as an imbalance between the growth and apoptosis of epithelial and stromal prostate cells leads to BPH [24]. Dysregulated apoptosis, driven by pro-apoptotic BCL-2 associated X (BAX) and anti-apoptotic BCL-2 proteins, induces BPH progression, with CASP3 playing a central role [25]. While in silico studies revealed CASP3 and BCL-2 among top 10 hub genes and pathway analysis demonstrated apoptosis as relevant, molecular changes in CASP3 and BCL-2 were insignificant, suggesting that paeonol’s alleviating effects against BPH progression are mainly associated with suppression of proliferation rather than enhancement of apoptosis.

Activation of Akt triggers many downstream pathways that enhance AR transcriptional activity, thereby accelerating BPH progression [26,27]. As PI3K-Akt pathways are involved in proliferation and apoptosis in prostate through crosstalk with AR, targeting PI3K-Akt signaling pathway appears to be a promising therapeutic target in prostate disease [26]. Akt1 was identified as the top 10 hub genes in PPI analysis, showed high binding affinity with paeonol of −6 kcal/mol, and KEGG pathway analysis highlighted PI3K–Akt signaling as relevant. In the BPH-induced rat model, paeonol treatment similarly inhibited Akt1 expression levels in the prostate tissue, which is consistent with previous studies of paeonol in prostate cancer cell lines [15]. Activation of Akt leads to activation or inhibition of many downstream targets regulating proliferation, cell growth, and apoptosis, such as cyclin D1 kinase, glycogen synthase kinase-3β (GSK3β), mammalian target of rapamycin (mTOR), and NF-κB [28]. As paeonol group showed marked molecular changes in genes related to proliferation, namely FGF1 and TGF-β1, regulation of Akt by paeonol treatment in BPH may lead to modulation in downstream related to proliferation rather than downstream pathways related to apoptosis. Taken together, paeonol may inhibit proliferation of prostate tissue by regulating local growth factors and inhibiting the PI3K–Akt pathway. Further investigation into the effects of paeonol on upstream pathways such as focal adhesion kinase (FAK)/PI3K and high mobility group box 1 (HMGB1)/PI3K is necessary to confirm these findings.

Inflammation is involved in the development of BPH, with BPH nodules largely comprising inflammatory cells [29]. Macrophages, B lymphocytes, and T cells in the prostate respond to pathogens and toxins, releasing proinflammatory cytokines, such as IL-1β and TNF-α. This chronic immune response further activates lymphocytes and macrophages, leading to cyclooxygenase-2 production and local hypoxia, thereby promoting BPH progression [30]. Among the many inflammatory cytokines, NF-κB and TNF may play pivotal roles in BPH progression as they promote proliferation and disrupt apoptosis in prostate cells [31]. To elaborate, NF-κB activates AR ligands independently Via androgen receptor variant 7 expression and thus induces hyperplastic growth [32,33], whereas TNF exacerbates NF-κB activation and macrophage-mediated inflammation [34]. The GO enrichment analysis suggested that paeonol primarily targets inflammatory pathways, whereas the KEGG pathway analysis indicated that its therapeutic effects may be associated with TNF, IL-17, and NF-κB signaling. Significant NF-κB suppression was observed in the paeonol-treated group in a dose-dependent manner, which is consistent with previous research findings that elucidated the anti-inflammatory properties of paeonol [13], while no significant changes were observed in TNF level. Collectively, paeonol may alleviate inflammation in BPH through regulation of NF-κB, and suppression of NF-κB may lead to inhibition of AR, thus downregulating the consequent transcription of proliferative genes.

As BPH is prevalent along with age, the production of free radicals, which trigger hyperplastic transformation in the prostate through oxidative stress, is also one of the factors leading to BPH progression [35,36]. Elevated levels of malondialdehyde and lipid peroxidation products, along with decreased levels of antioxidants, such as SOD and glutathione, are observed in patients with BPH [5]. GR, a key enzyme in the glutathione redox cycle, regulates oxidative stress by maintaining glutathione levels, and its dysregulation contributes to BPH progression. [37,38]. Paeonol 200 experimental group showed a significant reduction in GR level, whereas changes in SOD1 and CAT were unremarkable. Given that NF-κB inhibition leads to maintenance of glutathione levels [39,40], the observed decrease in GR level may be the consequence of NF-κB inhibition by paeonol.

Our study has some limitations. First, while DHT suppression is strongly suggested from the downregulated expression of 5AR2 and AR, direct quantification of DHT was not performed in the present study. Future studies of paeonol on its effects in prostate DHT levels are warranted for validation. Additionally, while our study elucidated many molecular mechanisms beneath improvements in BPH, future confirmation at protein levels would enhance comprehensive validation of the elaborated pathways. Lastly, although treatment periods of natural products in clinical studies of human BPH treatment typically range from 12 weeks to 96 weeks [10], the treatment period in this study was limited to 30 days, representing relatively short duration.

To the best of our knowledge, this is the first study to investigate the effects of paeonol on BPH using network pharmacology in conjunction with a testosterone-induced BPH rat model. As demonstrated in our study, certain in silico results differ from in vivo findings, underscoring the need for real-world validation. Nevertheless, network pharmacology provided valuable insights by enabling identification of potential therapeutic targets based on existing databases. Specifically, in silico analysis suggested involvement of PI3K-Akt and NF-κB pathways, and top 10 hub genes also included Akt and NF-κB. Consistent with these predictions, paeonol effectively inhibited mRNA expression of these genes in prostate tissue. Such modulations may lead to suppression of downstream proliferation and AR activation, thus ameliorating BPH progression as shown in the in vivo study. In the subsequent in vivo study, paeonol significantly reduced prostate volume and weight, which is noteworthy, as prostate volume is a strong predictor of BPH progression [7]. Oral administration of paeonol suppressed 5AR2 and AR expressions in the prostate tissue, suggesting a reduction in DHT production. Paeonol also attenuated prostatic proliferation by downregulating TGF-β1 and FGF-1, and epithelial hyperplasia was significantly mitigated in prostate histological images. Downregulation of Akt1 was elucidated, indicating inhibitory effects of paeonol on the PI3K–Akt pathway and the subsequent suppression of proliferation. Notable NF-κB regulation was also observed, suggesting alleviation of inflammation and subsequent inhibition of AR activity and the downstream transcription of proliferative factors. Collectively, this study revealed that paeonol exerts multifaceted therapeutic effects against BPH by suppressing DHT production, proliferation, and NF-κB signaling.

## 4. Materials and Methods

### 4.1. Network Pharmacology Analysis

#### 4.1.1. Identification of Paeonol and BPH-Associated Targets

The SMILES structure of paeonol (CC(=O)C1=C(C=C(C=C1)OC)O) was obtained from PubChem along with its two- and three-dimensional structures. To identify the potential targets of paeonol, various databases were used, including the Sea search server (https://sea.bkslab.org/, accessed on 13 July 2023), PubChem (https://pubchem.ncbi.nlm.nih.gov/, accessed on 13 July 2023), TCMSP (https://tcmspw.com/tcmsp.php, accessed on 13 July 2023), and Swiss Target Prediction (http://www.swisstargetprediction.ch/, accessed on 13 July 2023). These databases were utilized with filters set to “Homo sapiens” and probability values above 0. Several duplicate data entries were removed, and the target protein symbols were standardized using the UniProt protein database. The Swiss ADME database (http://www.swissadme.ch/, accessed on 13 July 2023) was used to evaluate drug likeness and adherence to Lipinski’s rules.

For the identification of BPH-associated genes, Genecards (https://www.genecards.org/, accessed on 13 July 2023) were used to discover genes related to BPH.

#### 4.1.2. Intersection Analysis of Paeonol and BPH Targets

To identify common targets between paeonol and BPH, VENNY 2.1.0 (https://bioinfogp.cnb.csic.es/tools/venny/index.html, accessed on 13 July 2023) was employed to analyze the intersections of these interactions and generate a Venn diagram.

#### 4.1.3. PPI Network Visualization

We constructed a PPI network to display the shared targets of paeonol and BPH using the STRING database (https://cn.string-db.org/, accessed on 13 July 2023). The interaction confidence threshold was set to “high confidence > 0.7”, with “Homo sapiens” as the selected organism. The network visualization excluded disconnected nodes, used “confidence” for the “meaning of edges”, and maintained default settings for all other parameters. The PPI network was visualized using the Cytoscape software (ver 3.9).

#### 4.1.4. Enrichment Analysis

GO and KEGG pathway enrichment analyses were conducted using the DAVID database (https://davidbioinformatics.nih.gov/, accessed on 13 July 2023). GO analysis categorizes gene functions into biological processes, cellular components, and molecular functions. KEGG analysis identified the significant pathways involved in BPH. Pathways related to BPH were visualized using dot plots generated in R Studio (ver 4.4.0), with the significance threshold set at *p* < 0.05.

#### 4.1.5. Molecular Docking Analysis

The docking targets were selected based on both network pharmacology predictions and their established roles in BPH pathophysiology. 5AR2 and AR were included due to their central involvement in dihydrotestosterone synthesis and androgen signaling. TGF-β1 and FGF-1 were chosen as representative growth factors implicated in stromal proliferation, extracellular matrix deposition, and epithelial hyperplasia. GR was included as a key antioxidant enzyme regulating redox balance, given the contribution of oxidative stress to BPH. In addition, Akt1 and NF-κB were selected as central signaling nodes mediating proliferation and inflammation, consistent with hub gene and pathway enrichment results. Collectively, these targets were prioritized as they reflect the multifactorial mechanisms underlying BPH progression. Molecular docking was performed to evaluate the binding affinity between paeonol and its targets, 5AR (PDB ID: 7BW1), AR (PDB ID: 5JJM), TGF-β1 (PDB ID: 3GXL), FGF-1 (PDB ID: 1FGK), GR (PDB ID: 1GSN), CAT (PDB ID: 1F4J), SOD1 (PDB ID: 1OZU), Akt1 (PDB ID: 3O96), BCL-2 (PDB ID: 1G5M), CASP3 (PDB ID: 3DEI), TNF (PDB ID: 5UUI), and NF-κB (PDB ID: 6POZ). The three-dimensional structures of these proteins were retrieved from the PDB database (https://www.rcsb.org/, accessed on 13 July 2023), and Biovia Discovery Studio Visualizer was used for structure preparation. Molecular docking was performed using AutoDock Vina (ver 1.2.0) via the PyRx (ver 0.8). For each protein, water molecules and nonessential atoms were removed, and polar hydrogens were added. Grid boxes were defined to fully encompass the reported ligand-binding pockets, with dimensions of 20–25 Å in each axis depending on protein size. The grid center was positioned at the coordinates of either the co-crystallized ligand or the documented active residues, such as ASP164/ASN160/ASN193 in 5AR2, GLU681/ARG752/LYS808 in AR, and PHE353/TRP178/CYS184 in NF-κB. Docking scores were calculated in kcal/mol, and the lowest-energy conformations were selected for further analysis. To further validate docking results, root-mean-square deviation (RMSD) values between paeonol docking poses and the native co-crystallized ligands were calculated. Most RMSD values were below 2.0 Å, indicating that the docking protocol produced reliable binding orientations. Ligand–receptor interaction maps were generated using Discovery Studio Visualizer, showing that paeonol consistently formed hydrogen bonds and hydrophobic contacts with key residues such as ASP164/ASN160 in 5AR2, GLU681/ARG752 in AR, and PHE353/CYS184 in NF-κB. In addition, to validate docking results, the binding affinities of paeonol were compared with those of reference ligands or known inhibitors, including finasteride, dihydrotestosterone, FGF-1, Akt1, and NF-κB. Although paeonol showed weaker affinities relative to high-potency inhibitors, as expected, it consistently established interactions with key residues, supporting its potential role as a modulator of BPH-related pathways.

### 4.2. In Vivo Study

#### 4.2.1. Paeonol Preparation Method

Paeonol (CAS RN: 552-41-0) was obtained from Tokyo Chemical Industry, Tokyo, Japan. The purity of paeonol exceeded 98%.

#### 4.2.2. Animal Study

Male Sprague–Dawley rats weighing 200–220 g were obtained from Orientbio Inc. (Seoul, Republic of Korea) and housed under a 12 h light/dark cycle with 40–70% humidity. They had unrestricted access to standard laboratory chow and water. Following a one-week adaptation period, the rats were randomly divided into five distinct groups (n = 5 in each group). In the NC group, rats underwent a sham operation without castration, received subcutaneous corn oil, and were orally administered phosphate-buffered saline (PBS) (5 mL/kg) daily. Except for the NC group, all rats were castrated and allowed to recover for one day before BPH induction. To induce BPH, castrated rats were subcutaneously injected with TP at a dose of 20 mg/kg in corn oil (Wako Pure Chemicals, Tokyo, Japan) for 30 days following castration. After castration, the rats were allocated into four groups and orally administered either PBS at 5 mL/kg (BPH group), finasteride (Merck and Co., Inc., Rahway, NJ, USA) at 1 mg/kg (Fin group), or paeonol at doses of 100 and 200 mg/kg (Pae100 and Pae200 groups, respectively) over a 30-day period.

#### 4.2.3. Body Weight and Prostate Measurement

Body weight was measured weekly throughout the experimental period. After 30 days, the rats were sacrificed, and their prostates were carefully excised, cleaned, and weighed. Prostate volume was calculated using the formula: prostate volume (cm^3^) = (a × b^2^)/2, where a is the longer and b is the shorter diameter. The ratio of prostate weight to body weight was also determined (mg prostate/100 g body weight).

#### 4.2.4. Histological Examination Using H&E Staining

The prostate tissue samples were fixed in 10% neutral-buffered formalin. The samples were dehydrated using a graded ethanol series (70%, 80%, 95%, and 100%) and embedded in paraffin to create tissue blocks. Sections of 4 μm thickness were cut from each block using a microtome and mounted on gelatin-coated slides. For staining, the slides were deparaffinized in xylene and rehydrated using a descending ethanol gradient (100%, 95%, 80%, and 70%), followed by distilled water. The tissue sections were stained with H&E. Digital images were captured using an optical microscope equipped with a high-resolution camera and further analyzed on a computer.

#### 4.2.5. Serum Testosterone Analysis

Blood samples were collected, coagulated at ambient temperature, and centrifuged at 3000× *g* for 20 min at 4 °C to obtain the serum. Serum testosterone levels were determined using a radioimmunoassay kit (Diagnostic Corporation, Los Angeles, CA, USA).

#### 4.2.6. Gene Expression Analysis of Prostate Tissues

qRT-PCR analysis was used to evaluate the expression of multiple target genes in prostate cells. Total RNA was extracted from prostate tissues, and 1 μg of RNA was reverse transcribed using Moloney murine leukemia virus reverse transcriptase in a reaction mixture containing PCR buffer, 5 mM MgCl2, 1 mM dNTPs, 20 U RNasin, and 2.5 μM Oligo(dT) primers. The reaction was obtained at 42 °C for 50 min, followed by 70 °C for 15 min. Glyceraldehyde 3-phosphate dehydrogenase (GAPDH) served as an internal control. Expression levels were quantified as fold changes by converting the threshold cycle data for each gene to relative quantification values against EF-1α, utilizing the SDS software 2.4. The specific primer sequences used for qRT-PCR analysis were as follows: 5AR1 (5′-GAGATATTCAGCTGAGACCC-3′ and 5′-TTAGTATGTGGGCAGCTTGG-3′); 5AR2 (5′-ATTTGTGTGGCAGAGAGAGG-3′ and 5′-TTGATTGACTGCCTGGATGGC-3′); AR (5′-TCCTGGCAGTCTTCAAGCC-3′ and 5′-GGAAGAGCACTGCTCTCAGG-3′); TGF-β1 (5′-AGCAACAATTCCTGGCGATAG-3′ and 5′-GTTGTTGCGGTCAATAGGTGA-3′); FGF-1 (5′-ATGGCTGCTGGAGAAGGATA-3′ and 5′-TGGAAGTGTTGAAGGGGAGA-3′); SOD1 (5′-GGTGGTGGCCAAAGGATGAAG-3′ and 5′-CAGGTCTCCAACATGCCTCTCT-3′); CAT (5′-AGGTCAGGAGAAACGGGAG-3′ and 5′-TCTCCAGACCTGCGAAAGC-3′); GR (5′-TGACCGAGTTTGGGATGGAG-3′ and 5′-CCACGTTGTTCTCCTCCTTG-3′); AKT1 (5′-TGAAGGAGGGAGTGTGTGGA-3′ and 5′-GGAGAGGAGGAGGAAGGTGT-3′); BCL-2 (5′-GGTGAACTGGGGGAGGATTG-3′ and 5′-TCACTTGTGGCCCAGATAGG-3′); CASP3 (5′-TGCAGGAGCTGTTTCAGAG-3′ and 5′-GTGAGGAACTGAAAGGAAAG-3′); TNF-α (5′-AGCCGATGGGTTGTACCTTG-3′ and 5′-CTGGTAGGAGACGGCGATGA-3′); NF-κB (5′-CTGGTGCCTGTTGTGTGAA-3′ and 5′-TGGCTGTGGGGTAGGAGGTA-3′); GAPDH (5′-TGCCAAGGCTGTGGGCAAGG-3′ and 5′-GCTTCACCACCTTCTTGATG-3′).

#### 4.2.7. Liver and Kidney Function Assessment

Biochemical markers, including blood urea nitrogen (BUN), creatinine, aspartate aminotransferase (AST), and alanine aminotransferase (ALT), were measured to assess liver and kidney functions.

#### 4.2.8. Statistical Analysis

Statistical analyses were performed using the GraphPad Prism software (version 5.0). Differences between groups were evaluated using a one-way analysis of variance, followed by Tukey’s post hoc test. Data are presented as means ± standard errors of the mean, and a two-tailed *p* < 0.05 was considered statistically significant. Significance levels are denoted by asterisks (*), where one asterisk indicates *p* < 0.05, two asterisks indicate *p* < 0.01, and three asterisks indicate *p* < 0.001.

## 5. Conclusions

This study investigated the multifaceted effects of paeonol on BPH, including its regulation of DHT production, growth factors, apoptosis, inflammation, and oxidative stress. Following the identification of potential therapeutic targets with network pharmacology, validation was performed using a BPH-induced rat model. Mechanisms involved in DHT regulation through inhibition of 5AR2 and AR, and suppression of proliferation Via downregulation of TGF-β1 and FGF-1 were highlighted. Additionally, paeonol’s effects on regulating apoptosis, inflammation, and oxidative stress through the suppression of AKT1, NF-κB, and GR were also elucidated. These mechanistic pathways contributed to remarkable reduction in the prostate volume and histological improvement in the paeonol-treated BPH rat model. Collectively, these findings highlight the potential use of paeonol for BPH treatment. Future investigations on upstream regulatory pathways, and validation at protein levels are warranted for future research.

## Figures and Tables

**Table 1:** Paeonol–target binding affinity.

Target Name	PDB ID	Binding Affinity (kcal/mol)
5AR2 (5α-reductase type 2)	7BW1	−5.9
AR (Androgen receptor)	5JJM	−6.7
TGF-β1 (Transforming growth factor beta-1)	3GXL	−6.5
FGF-1 (Fibroblast growth factor 1)	1FGK	−6.0
SOD1 (Superoxide dismutase 1)	1OZU	−5.3
CAT (Catalase)	1F4J	−5.2
GR (Glutathione reductase)	1GSN	−6.1
AKT1 (Alpha serine/threonine-protein kinase)	3O96	−6.0
BCL-2 (B-cell lymphoma 2)	1G5M	−5.3
CASP3 (Caspase 3)	3DEI	−5.1
TNF (Tumor necrosis factor)	5UUI	−5.3
NF-κB (nuclear factor-κB)	6POZ	−6.5

## Data Availability

Data presented in this study is contained within the article. Further inquiries can be directed to the corresponding authors.

## Abbreviations

The following abbreviations are used in this manuscript:

5AR2	5α-reductase 2
AGE–RAGE	Advanced glycation end-product receptors for advanced glycation end products
Akt1	Alpha serine/threonine-protein kinase
AKT	Protein kinase B
ALT	Alanine aminotransferase
AR	Androgen receptor
AST	Aspartate aminotransferase
BCL-2	B-cell lymphoma 2
BPH	Benign prostatic hyperplasia
BUN	Blood urea nitrogen
CASP3	Caspase-3
CAT	Catalase
COX-2	Cyclooxygenase-2
DHT	Dihydrotestosterone
FAK	Focal adhesion kinase
FGF-1	Fibroblast growth factor 1
GAPDH	Glyceraldehyde 3-phosphate dehydrogenase
GR	Glutathione reductase
GSK3β	Glycogen synthase kinase-3 beta
H&E	Hematoxylin and eosin
HMGB1	High mobility group box 1
IL	Interleukin
LUTS	Lower urinary tract symptoms
mTOR	Mammalian target of rapamycin
NF-κB	Nuclear factor-κB
PBS	Phosphate-buffered saline
PI3K	Phosphatidylinositol 3-kinase
PPI	Protein–protein interaction
qRT-PCR	Quantitative real-time polymerase chain reaction
ROS	Reactive oxygen species
RMSD	root-mean-square deviation
SOD1	Superoxide dismutase 1
TGF-β1	Transforming growth factor beta-1
TNF	Tumor necrosis factor
